# Editorial: Perspectives of Astrocytes in Neurodevelopmental and Neurodegenerative Diseases: From Mechanistic Studies to Therapeutic Applications

**DOI:** 10.3389/fncel.2022.857229

**Published:** 2022-02-16

**Authors:** Egor Dzyubenko, Dirk M. Hermann, Junhui Wang

**Affiliations:** ^1^Department of Neurology and NeuroScience Lab, University Hospital Essen, Essen, Germany; ^2^Sinai Health System, Lunenfeld-Tanenbaum Research Institute, Mount Sinai Hospital, Toronto, ON, Canada

**Keywords:** neuropathology, reactive astrocytes, gliovascular unit, neuron-glia interactions, neurotoxic astrocyte, hepatic encephalopathy (HE), temporal lobe epilepsy, neuronal plasticity

Neurodevelopmental and neurodegenerative diseases including early-onset epilepsy, dementia, and stroke are major challenges for modern medicine because their pathogenesis is not yet entirely understood. Brain neurons are controlled by astrocytes, and astrocytes are critical for neuronal survival and function (Hermann and Chopp, 2012). Investigating the role of astrocyte-mediated mechanisms is therefore pivotal for understanding pathophysiological processes in the injured brain and developing novel therapeutic strategies.

Astrocytic alterations are commonly associated with neuroinflammation (Hermann et al., 2021). Changes in astrocyte polarization strongly influence neuronal survival, for example, via neurotransmitter release dysregulation (Acioglu et al., 2021). Reactive astrocytes have a major impact on neuronal activity in temporal lobe epilepsy, Twible et al. highlight in their review within this Research Topic. Temporal lobe epilepsy is associated with aberrant mossy fiber sprouting and hippocampal sclerosis that are frequently accompanied by reactive astrogliosis. The authors summarized results on molecular signaling pathways, cellular histological alterations, and clinical pathological observations that indicate the role of astrocytes in mossy fiber sprouting in temporal lobe epilepsy. Seizures induce the expression of synapse formation proteins in astrocytes, which facilitates axonal sprouting. The original research article by Yu et al. further supports this evidence showing that the non-competitive N-methyl-D-aspartic acid receptor (NMDA-R) antagonist MK-801 induces a transient increase in BDNF expression by pathways involving NMDA-R–PI3K–ERK signaling. These findings suggest that altered astrocytic BDNF signaling to hippocampal neurons contributes to the acute changes in behavioral states and may be involved in the pathogenesis of psychosis. Harnessing neuroplasticity-promoting properties of astrocytes is promising for developing novel therapeutic approaches in regenerative medicine. The review by Zhao et al. provides a critical update on glial cell-based and transplantation therapies in restoring the neurovascular unit function in neurodevelopmental and neurodegenerative disorders. Recent data highlighted the endogenous reparative potential of glial cell-based therapy (Jiang et al., 2021), and transplantation of pre-differentiated neural and vascular cells (Zhang et al., 2020) has been proposed to ameliorate neurological symptoms by maintaining brain homeostasis.

Homeostatic regulation of neuronal excitability requires ionic buffering and glutamate uptake by astrocytes. Using calcium imaging, Khezerlou et al. investigated the negative feedback role of astrocytes in shaping excitation in neuronal-astrocytic co-cultures with low (co-cultures containing 50% astrocytes) and high (co-cultures containing >70% astrocytes) negative feedback. The authors show that astrocytes can influence both calcium signaling regulation and nitric oxide production in multicellular neuron-glia interactions.

Recent evidence indicates that immune responses play a central role in ischemic brain injury and stroke recovery (Hermann et al., 2021). In this Research Topic, Pan et al. explore the involvement of perforin, a glycoprotein that is responsible for pore formation in cell membranes, in the interaction between immune cells and astrocytes post stroke. The authors show that perforin is expressed by CD8^+^ T cells, NK cells, NKT cells, and microglia after ischemic brain injury, which correlates with the enrollment of neurotoxic astrocytes. Genetic ablation of perforin in Prf1^−/−^ mice reduced neurotoxic astrogliosis, infarct volume, and neurological deficits after ischemic stroke. These findings indicate that perforin is a novel target for restorative therapy in stroke.

Astrocytes also play a role in neurodegeneration that is not induced by brain damage, Jin et al. show using an *in vitro* model of hepatic encephalopathy. Elevation of ammonia levels in hepatic encephalopathy induces neurotoxicity in astrocytes. The authors reveal that hydrogen sulfide (H_2_S) protects against ammonia-induced neurotoxicity by activating Nrf2/ARE signaling in astrocytes. In patients with hepatic encephalopathy, blood plasma levels of H_2_S and the presence of H_2_S-producing bacteria in gut microbiota negatively correlated with the severity of encephalopathy.

Taken together, this Research Topic highlights novel mechanisms of astrocyte-mediated disturbances of neuronal function. Astrocytes play pivotal roles in the pathophysiology of neurodevelopmental and neurodegenerative disorders, as shown in a variety of *in vitro* and *in vivo* disease models in the past and further demonstrated within this Research Topic. Besides others, this Research Topic provided new signal mechanisms, via which regulatory functions of astrocytes are controlled under conditions including epilepsy, ischemic stroke, and metabolic encephalopathy. The emerging experimental data indeed supports clinical significance of astrocyte-neuronal communication in a large variety of disease contexts, and we believe that advances in the field will provide promising therapeutic targets, via which restorative processes can be enhanced in the injured brain.

## Author Contributions

All authors contributed equally by discussing and summarizing the results, drafting the manuscript, and making final corrections.

## Funding

This work was supported by the German Research Foundation (projects 405358801 and 389030878 to DH) and (project 467228103 to ED) and Federal Ministry of Education and Science (project 161L0278B to DH).

## Conflict of Interest

The authors declare that the research was conducted in the absence of any commercial or financial relationships that could be construed as a potential conflict of interest.

## Publisher's Note

All claims expressed in this article are solely those of the authors and do not necessarily represent those of their affiliated organizations, or those of the publisher, the editors and the reviewers. Any product that may be evaluated in this article, or claim that may be made by its manufacturer, is not guaranteed or endorsed by the publisher.
